# Use of proximity ligation shotgun metagenomics to investigate the dynamics of plasmids and bacteriophages in the gut microbiome following fecal microbiota transplantation

**DOI:** 10.1080/19490976.2025.2559019

**Published:** 2025-09-15

**Authors:** Samuel Bryson, Zachary Sisson, Bradley Nelson, Jonas Grove, Emily Reister, Ivan Liachko, Benjamin Auch, Carolyn Graiziger, Alexander Khoruts

**Affiliations:** a Phase Genomics, Seattle, WA, USA; b Department of Medicine, Division of Gastroenterology, Hepatology, and Nutrition, University of Minnesota, Minneapolis, MN, USA; c Center for Immunology and BioTechnology Institute, University of Minnesota, Minneapolis and St. Paul, USA

**Keywords:** Proximity ligation shotgun metagenomics, *Clostridioides difficile*, fecal microbiota transplantation, antimicrobial resistance genes

## Abstract

Proximity ligation shotgun metagenomics facilitate the analysis of the relationships between mobile genetic elements, such as plasmids and bacteriophages, and their specific bacterial hosts. We applied this technique to investigate the changes in the fecal microbiome of patients receiving fecal microbiota transplantation (FMT) for recurrent *Clostridioides difficile* infections (rCDI). FMT was associated with successful engraftment of donor bacteria along with their associated bacteriophages. While fecal microbial diversity increased in all patients, the extent of specific bacterial taxa engraftment varied among individual patients. Interestingly, some donor bacteriophages remained closely linked to their original bacterial hosts, while others expanded their associations across different bacterial taxa. Notably, FMT partially reduced the content of vancomycin resistance and extended-spectrum beta-lactamase genes in the fecal microbiome of rCDI patients.

## Introduction

The growing appreciation of the gut microbiota as an organ-like structure that performs multiple vital functions for the host^1^ has led to investigations of restorative treatment strategies for conditions associated with gut microbiota injury or dysfunction. Fecal microbiota transplantation (FMT) has emerged as a crude but highly effective approach to breaking the cycle of recurrent *Clostridioides difficile* infections (rCDI).^2^ Microbiota transplant-based strategies are now being explored for numerous other indications, including inflammatory bowel disease,^3–6^ optimization of cancer checkpoint immunotherapy,^7,8^ complications of intensive chemotherapy and hematopoietic stem cell transplantation,^9,10^ advanced liver disease,^11,12^ autism,^13^ and others.

Over the past decade, FMTs have evolved into increasingly standardized formulations. FMT differs from traditional therapeutics, such as small-molecule chemical compounds and protein biologics, in their extraordinary complexity and the uncertainty surrounding their mechanisms of action.^14^ Thus, the pharmacokinetics and pharmacodynamics of FMT require high-throughput nucleic sequencing and computational technologies along with integration of multi-omics data, such as metabolomics, immune profiling, nutritional input, and clinical parameters.

In the case of rCDI, multiple studies have demonstrated that FMT results in restoring the bacterial community to the structure that resembles the donor. Clinical success correlates with increases in the bacterial families Lachnospiraceae, Ruminococcaceae, and Bacteroidaceae, mainly driven by donor strains.^15,16^ Therefore, one major area of investigation aimed at improving FMT outcomes has been enhancing the engraftment of donor bacterial strains.^17,18^ The recovery of antibiotic-depleted bacteria restores secondary bile acid metabolism and certain short-chain fatty acids, which enhance colonization resistance against *C. difficile* bacteria.^14^ However, additional anti-CDI mechanisms, such as donor-derived bacteriophages, bacteriocins, and activation of host immune mechanisms, have been suggested to play potentially important roles.^14^

**Figure 1.**
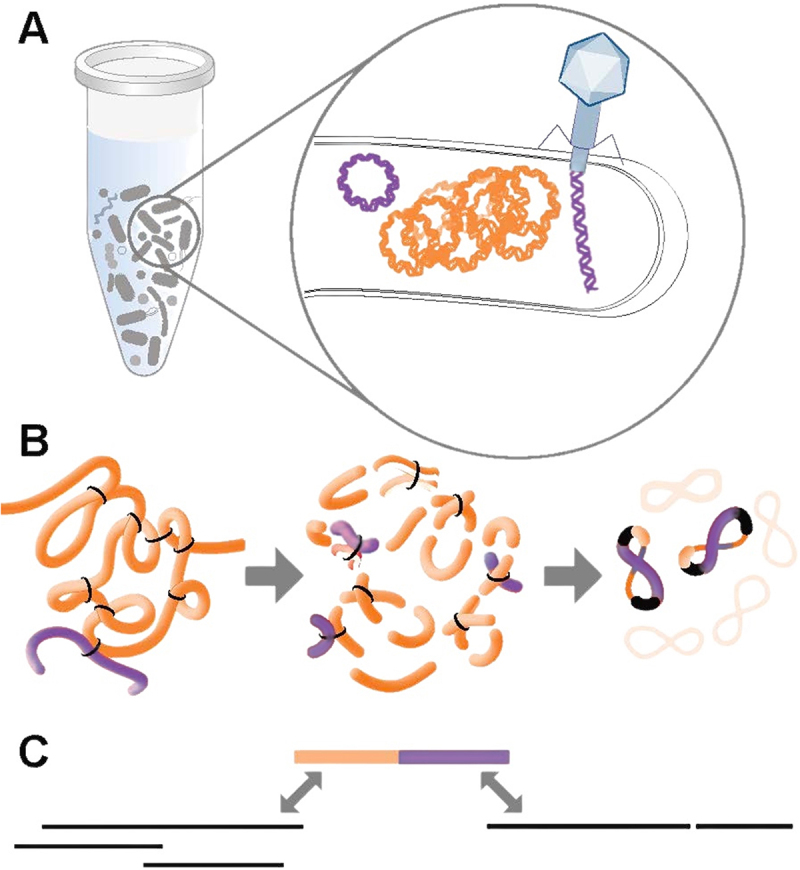
Proximity guided metagenomics links mobile elements to hosts. The graphic shows the major steps involved in the biochemical methodology. (A) The sample contains contact microbial cells along with mobile elements (plasmids and active phages) in close proximity to the host chromosome. (B) First, proximity ligation crosslinks neighboring dsDNA within intact cells using formaldehyde. Next (arrow), cells are lysed and dsDNA is digested with restriction enzymes. After that (arrow), the crosslinked molecules are end-joined and paired-ended libraries are created. (C) Alignment of paired end reads links assembled contigs of mobile elements and host microorganisms.

Recently, an increasing number of investigations employed shotgun metagenomics to explore changes in the gut microbiome beyond bacterial populations, including shifts in bacteriophage populations.^19–23^ Bacteriophages and plasmids comprise a numerically highly abundant component of the gut microbial ecosystem, playing critical roles in its resilience and functionality. Some of their genes encode products that support and modulate bacterial metabolism.^23^ They may also carry genes for antibiotic resistance and virulence, a particular concern for both developers of LBPs and regulators.^24–26^ Importantly, the functional and pathogenic potentials of bacteriophages and plasmids depends in part on their specific bacterial hosts. However, identification of pairings with specific bacterial hosts using shotgun metagenomics is generally based on indirect sequence-based evidence, which has limited sensitivity and specificity.^27,28^ Proximity ligation shotgun metagenomics solve this problem by establishing a physical linkage between bacterial hosts and extrachromosomal elements using formaldehyde fixation *prior to further sample processing* and DNA sequencing (Figure 1).^29–31^

In this study, we applied proximity ligation metagenomics to a cohort of 30 rCDI patients who received FMT using a standardized, encapsulated fecal microbiota preparation derived from a single donor. Fecal samples were collected before and after treatment to assess the degree of restoration of patient gut microbiomes, examine the engraftment of donor bacteria as well as bacteriophages and plasmids, and investigate the impact on the overall AMR carriage.

## Results

### Capsule FMT restores bacterial and bacteriophage diversity in the fecal microbiome

After failing multiple rounds of antibiotics against *C. difficile*, patients were treated with a single oral dose of encapsulated, freeze-dried microbiota from a healthy donor, leading to a clinical cure in 27 out of 30 patients (their clinical metadata and demographics are included in the Supplementary Table S1). Proximity ligation metagenomics was applied to the donor sample plus 25 pre-FMT fecal samples, and 18, 18, 3, and 19 fecal samples collected at 1, 2, 3, and 4 weeks post-FMT. The assembly and binning of these 84 metagenomes yielded an average assembly length of 276,245,467 bases (±161,637,900 S.D.) and 202 (±164 S.D.) metagenome assembled genomes (MAGs) that account for an average of 86.3% (±9.15% S.D.) of the assembled length in each sample (Supplementary Table S2).

**Figure 2.**
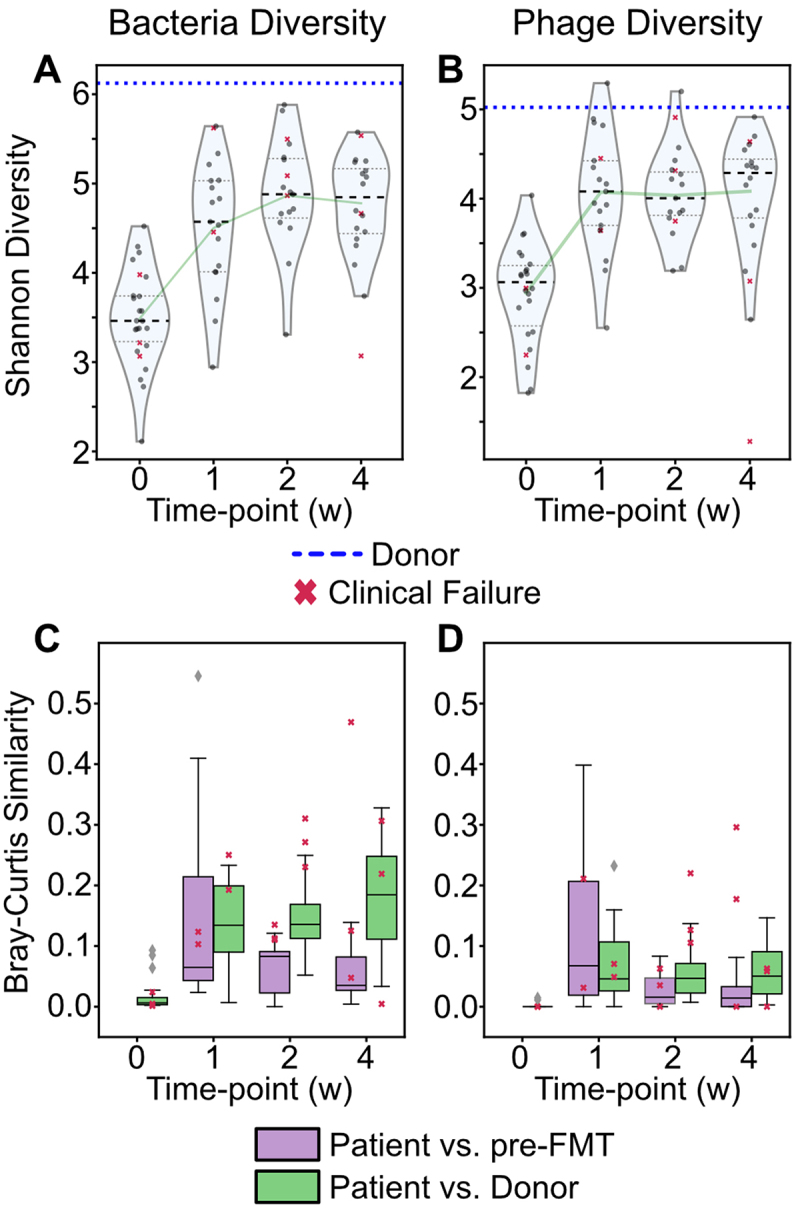
Diversity of bacteria and phages across the FMT cohort. Alpha-diversity of (A) Bacteria and (B) phages in pre-FMT (time point 0) and post-FMT (time points 1–4 weeks) samples compared to the donor (dotted blue line). Beta-diversity comparisons for bacteria (C) and phages (D) between patient samples at each time point and the corresponding pre-FMT (purple) and donor (green) samples.

To compare bacterial and phage diversity among timepoints, the recovered microbial and phage MAGs were clustered into operational taxonomic units (bOTUs and vOTUs, respectively, see Materials and Methods for details). As expected, the bacterial alpha-diversity of pre-FMT microbial populations was lower relative to that of the donor but increased post-FMT (Figure 2(A)). Interestingly, as seen previously,^32,33^ the bacterial alpha-diversity of post-FMT samples never reached that of the donor (Shannon Diversity Index = 6.1), suggesting possible host- or FMT route-associated engraftment constraints or incomplete viability of some donor bacterial taxa in this formulation.

The alpha-diversity of phages followed the same trend observed for microbes (Figure 2(B)). However, the phage Shannon diversity calculated for some post-FMT samples was near or greater than that observed for the donor sample (Shannon diversity index = 5.0). Patient pre- and post-FMT timepoints had significantly different phage-diversity (ANOVA, *p* = 1.4 × 10^−8^) and the mean post-FMT phage alpha-diversity was significantly higher than pre-FMT (adj. *p* < 0.01, Tukey’s HSD test). The post-FMT phage alpha-diversity remained stable within the sampled timeframe of individual recipients.

### The post-FMT bacterial and bacteriophage communities become more similar to the donor after one week

Next, we compared the similarity (Bray–Curtis Similarity, see Methods) between each patient’s post-FMT sample and the donor or corresponding pre-FMT sample. Bacterial beta diversity (Figure 2(C)) indicated that pre-FMT samples were significantly different from the donor sample (adj. *p* < 0.01, Tukey’s HSD test). Although at week 1 there was not a significant difference from the donor or pre-FMT samples in the bacterial communities (adj. *p* = 1.0, Tukey’s HSD test), week 2 and 4 samples were significantly different from the donor and pre-FMT samples (adj. *p* < 0.01, Tukey’s HSD test). This result is consistent with the previously described pharmacokinetics of our capsule FMT formulation, where more than week was required to observe the greatest abundances of donor bacteria in rCDI patients.^33^

Similar to the changes seen in bacterial communities following FMT treatment, there was greater similarity of phage communities in post-FMT samples to the donor sample than in the pre-FMT samples (Figure 2(D)). However, the shift toward the donor phage composition was less pronounced, and there were no significant differences in similarity to the donor and pre-FMT phage communities observed at week 1, 2, or 4 (adj. *p* = 0.75, 0.95, and 0.72, respectively, in Tukey’s HSD test).

### Microbiome engraftment patterns are patient-specific

**Figure 3.**
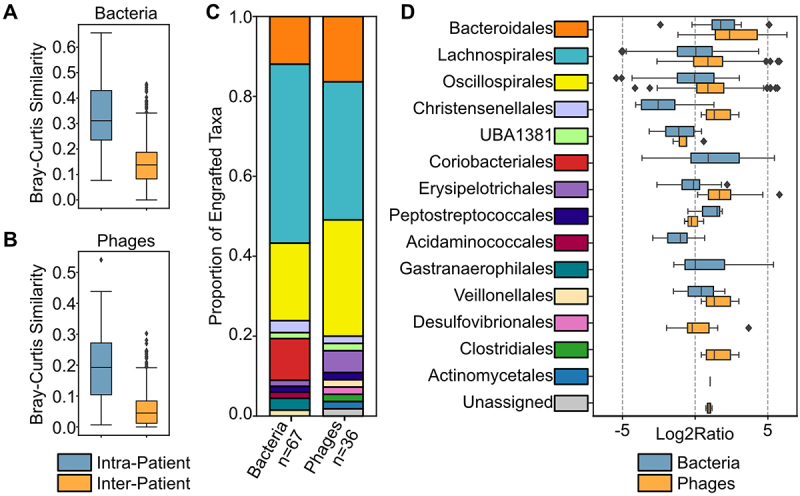
Engraftment of bacteria and phages. Beta-diversity comparisons between post-FMT samples for (A) bacteria and (B) phages. (C) Taxonomic distribution of engrafted bOtus and vOtus. (D) Log2 ratio of relative abundances, post-FMT vs. donor, observed for engrafted bOtus and vOtus.

Interestingly, engraftment of donor bacteria and bacteriophages exhibited patient-specific outcomes over time. Beta diversity comparisons revealed that bacterial (Figure 3(A)) communities were significantly more similar within patient post-FMT samples than between patients. Phage communities (Figure 3(B)) were also significantly more similar within patient samples than between patients. Since all FMT product was prepared from a single donor, these results can be attributed to recipient-associated factors, such as the existing recipient microbiota and diet.^34^

To further examine differences in bacterial and phage engraftment, we identified 67 bOTUs and 36 vOTUs that were present in the donor sample and identified in one of the more post-FMT samples. The majority of these bOTUs and vOTUs were assigned to the orders Lachnospirales (*n* = 30 and 19 patients, respectively), Oscillospirales (*n* = 13 and 16 patients, respectively), and Bacteroidales (*n* = 8 and 9 patients, respectively) (Figure 3(C)). In line with the beta diversity comparisons among and between patients, the observed abundances for the engrafted bOTUs and vOTUs exhibited a high degree of variability (Figure 3(D)). For example, the relative abundances observed for the most frequently engrafted bOTU (#344, *n* = 19 samples) assigned to Bacteroidales varied by a factor of between 0.19 and 9.0 times its relative abundance in the donor sample. The most frequently observed Bacteroidales phage (#1328, *n* = 9 samples) varied in abundance between 6.2 and 79 times its relative abundance in the donor sample. Variation was even greater for bOTUs assigned to Lachnospirales and Oscillospirales and their associated vOTUs. Lachnospirales mOTUs and their vOTUs varied in relative abundance between 0.030– and 21 and 0.16 and 56 times their donor sample abundances, respectively. Oscillospirales mOTUs and vOTUs varied in abundance by a factor of 0.023–8.2 and 0.057–51, respectively.

### Donor phages engraft and exhibit expanded host ranges

**Figure 4.**
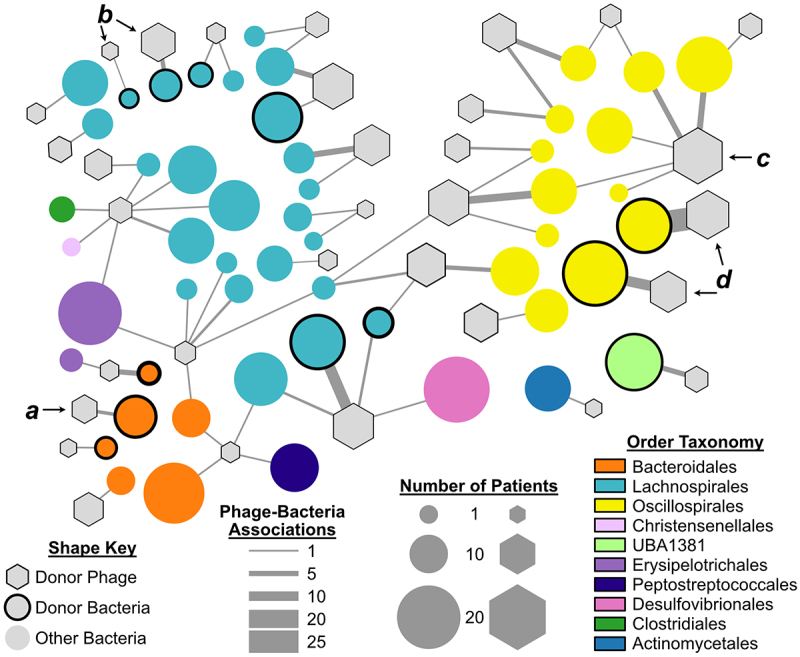
Graph representation of donor phage engraftment. The number of interactions detected between donor phage (vOtus, hexagons) and bacteria (mOtus,circles) is indicated by the edge weight. The order level assignment of bacteria is indicated by the color key. The number of patients vOtus and bOtus detected is indicated by the node size. Specific interactions discussed in the text are indicated by *a*, *b*, *c*, and *d*.

Next, we investigated the frequency and diversity of detected interactions between engrafted phages and corresponding bacterial hosts (Figure 4). Donor phage–bacterial interactions captured using the DNA ligation method were detected for 32 donor-derived vOTUs in post-FMT samples. Some of the original phage–bacterial host interactions seen in the donor maintained their exclusivity in the recipient patients. For example, interactions between the previously discussed *Bacteroidales* phage (vOTU #1328) and host bacterium (bOTU #344) were specific (Figure 4(a)). However, most phages that engrafted were linked to bacteria that were not present in the donor material. Thus, the donor phages found new hosts in the recipients.

Among the diverse set of engrafted Lachnospirales phages, exclusive phage–bacteria interactions were only observed for two of the engrafted phages (Figure 4(b)), while the majority of proximity linkages indicated associations with bacteria not detected in the donor sample. This trend was also observed among engrafted Oscillospirales phages. Proximity linkages between the most frequently observed of these (vOTU #157, *n* = 17 patients) were observed between five different bOTUs (Figure 4(c)), but most often with the ubiquitous gut microbe *Evtepia gabavorous* (bOTU #425, *n* = 6 interactions). Two Oscillospirales phages (Figure 4(d)) were detected among multiple patients, but interactions were exclusive with the donor host bacteria.

### FMT for rCDI is only partially effective in depleting clinically important AMR genes

**Figure 5.**
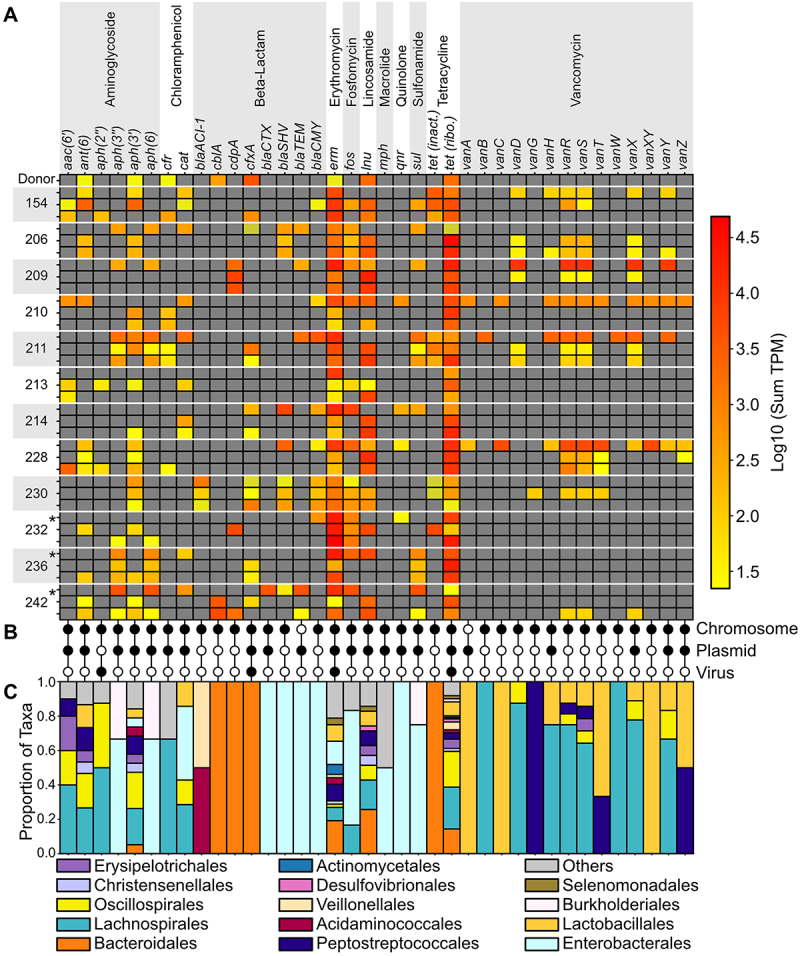
Abundance and distribution of clinically relevant AMR genes. The heatmap (A) depicts the sum of abundances for bacteria harboring individual AMR genes for each patient where a pre-FMT plus 2-week and 4-week post-FMT samples were available, corresponding to the three rows depicted for each patent indicated on the left axis. Color intensity indicates the sum of bacteria TPM values in each sample (log10 transformed). “*” indicates three clinical failures. AMR genes are listed individually, or where appropriate combined such as with *erm* and *tet* genes. A full list of genes and aggregations is available in Supplementary Table S3. (B) Genomic sources for each observed AMR gene. (C) Taxonomy of bacteria hosts for each AMR gene.

Multiple studies have shown that FMT has the potential to decrease the overall burden of AMR genes, which are present in greater abundance in rCDI patients relative to healthy individuals.^35–42^ Here, we examined the genomic, phage, and plasmid carriage of AMR genes in donor, pre-FMT, and post-FMT samples (Figure 5).

Across all FMT trial patient and donor samples, 126 AMR genes were identified and assigned to bacterial genomes or mobile elements. Of these, 75 were linked to high-quality bacterial MAGs as chromosomal, plasmid, or phage genes, ~83%, ~16%, and ~1%, respectively. Not surprisingly, donor samples contain some AMR genes, which are ubiquitous in the environment and across human populations.^29,43^ Thus, all donor and recipient samples contained *erm* (ribosomal methyltransferase) and *tet* (tetracycline-resistant ribosomal protection) genes, and most contained *lnu* (lincosamide nucleotidyltransferase) genes. The ubiquity of *erm* and *tet* genes was further demonstrated by their detection in chromosomes, plasmids, and phages, as well as the diversity of bacterial taxa that harbor these genes (Figure 5).

Including the widely distributed *erm*, *tet*, and *lnu* genes, engraftment of bacterial taxa with AMR genes was rare; only ~4% of the total AMR gene identifications across all 84 metagenomes were associated with engrafted taxa. The donor sample included on bOTU assigned to *Bacteroides uniformis* that contained the class A beta-lactamase gene *cblA*. This bacterial genome was identified in one patient (235) at 2 and 4 weeks post-FMT. The *cblA* gene, also identified in *B. uniformis* bOTU, was found in patient 242 and patient 216 post-FMT samples, but these MAGs were not confidently associated with the donor strain. Similarly, the *cfxA* class A beta-lactamase gene was linked to a donor strain assigned to *Prevotella copri*, which was also identified in patients 211 and 216 post-FMT samples. However, a nearly identical strain was also found in the patient 42 pre-FMT metagenome, confounding any conclusive determination for the source of this *P. copri* strain. Notably, global surveys have identified both of these beta-lactamases in the majority of stool samples in strict association with typically nonpathogenic bacteria in the order *Bacteroidales*.^44^

Because vancomycin is the standard treatment for *C. difficile* infections, we also sought to characterize the presence and potential persistence of vancomycin resistance following treatment. Plasmids carrying the *vanA* resistance cassette (*vanRSHAXYZ*) were associated with *Enterococcus* species in the pre-FMT samples for patients 210 and 228 but were no longer detected following FMT (Figure 5). Plasmids carrying the *vanA* gene in association with *Enterococcus* were also noted in the pre-FMT and 1-week post-FMT samples of patient 233; however, we did not have sufficient DNA for analysis in subsequent samples. A chromosomal *vanA* gene was identified in the *Paenibacillus dendritiformis* genome in patient 224. The *vanB* cassette (*vanRSYWHBX*) was identified on the chromosome of an *Enterocloster clostridioformis* bOTU that was present in the pre-FMT sample for patient 211 (Figure 5) and in patient 233 (pre- and 1-week post-FMT). The *vanC* cassette (*vanCXYTRS*) was identified in two bOTUs assigned to *Enterococcus gallinarum* and *E. casseliflavus*, respectively. The *E. gallinarum* bOTU containing *vanC* was found in pre-FMT samples for patients 210, as well as 221 and 228. *E. casseliflavus* containing *vanC* was identified in patients 183, 217, 224, 228, and 42. A *C. difficile* bOTU was identified that contained *vanG* and associated genes (*vanR*_*Cd*_, *vanS*_*Cd*_, *vanZ1*_*Cd*_, *and vanT*_*Cd*_) were found in pre-FMT samples for patients 262 and 154.

Extended spectrum β-lactamases (*ESBL*) were exclusively detected in MAGs and plasmids associated with the order Enterobacterales. SHV genes (Figure 5) were typically identified as chromosomal genes on MAGs assigned to *Klebsiella pneumoniae*. The one exception (not depicted in Figure 5) was identified on a plasmid associated with *K. pneumonia* in patient 233. *TEM-1* genes that were assigned to a host bacteria were typically identified on plasmids of *E. coli*, with the exception of a plasmid assigned to *Morganella morganni* in the pre-FMT sample of patient 211. *CTX-M* genes were identified in four patients pre-FMT samples (233, 242, 246, and 254) and two post-FMT samples (183 and 233). These genes were identified on the chromosomes of *Kluyvera* species and on a single plasmid linked to *E. coli* in patient 233. The cephalomycinase (*CMY*) gene was most commonly identified in MAGs or plasmids assigned to *Citrobacter* species but was associated with the chromosomes of *E. coli* and *K. pneumonia* in patients 211 and 230 pre-FMT samples, respectively. In some patients, e.g., 206, 209, 211, 242, FMT was associated with loss of detection or marked reduction in density of the plasmid encoded *TEM-1* genes (Figure 5). However, FMT had only variable impact on the presence of chromosome encoded *SHV* and *CMY* genes (Figure 5).

## Discussion

Mobile genetic elements (MGEs), including plasmids and bacteriophages, are major drivers of bacterial evolution. They allow bacteria to rapidly acquire new genetic traits, leading to rapid adaptation and emergence of attributes like antibiotic resistance and virulence factors. All FMT-based formulations contain MGEs, which may contribute to their therapeutic benefit but also pose a risk by accelerating the spread of harmful genes that may represent a threat to public health. On the other hand, FMT has also been shown to decrease the burden of AMR genes, many of which are encoded on plasmids and bacteriophages.^35,36,45^ To better understand the functional and pathogenic roles of these extrachromosomal elements, it is essential to identify the bacterial species with which they are associated. In our study, we utilized proximity ligation shotgun metagenomics to analyze the microbiome changes in a cohort of rCDI patients treated with a standardized oral capsule FMT formulation.

As we reported previously, we observed restoration of bacterial diversity within the initial weeks following oral capsule FMT.^46^ In addition, our current findings demonstrate that the engraftment of donor bacteria is accompanied by donor bacteriophages, even though the FMT preparation used was composed of a purified microbial fraction from stool. This finding is not surprising since most bacteriophages in the human gut are temperate and integrated into bacterial genomes as prophages.^47,48^ Some donor bacteriophages remained strictly associated with donor bacterial hosts. However, in many cases, the bacteriophages formed connections with new bacterial hosts that were not found in the donor, suggesting that FMT may trigger activation of donor prophages. While additional corroborative investigations would be needed, this observation supports the idea that bacteriophages could have a therapeutic role in treating rCDI through FMT, a hypothesis that was suggested by the apparent efficacy of sterile fecal filtrate^49^ and a correlation between higher donor bacteriophage α-diversity and successful FMT outcomes.^21^ Interestingly, while the overall pattern of microbiome restoration in rCDI patients was associated with an increased bacterial α-diversity, the engraftment of specific donor taxa varied considerably among individual patients. This variability likely stems from differences in the residual indigenous microbiota at the time of FMT, as well as various host-related factors, such as diet, medications, and medical co-morbidities. Generally, donor bacteriophages followed their bacterial hosts.

Similarly to prior studies of healthy individuals,^29,50^ donor microbiota contained many AMR genes. However, the donor AMR genes are widely found in the human gut across the world. Class A/B beta-lactamases and tetracycline and lincosamide resistance genes were reported to be present even in Hadza hunter-gatherer populations, which had little prior antibiotic exposure.^43^ Importantly, none of these genes were associated with common opportunistic clinical pathogens, which include members of the Enterobacteriaceae and Enterococcaceae families. The donor microbiota did not contain genes for extended spectrum beta-lactamases or any vancomycin resistance genes.

Patients that develop rCDI often have a high burden of antibiotics leading up to their initial infection, which is then followed by repeated cycles of vancomycin in attempts to eradicate *C. difficile*. Thus, it was not surprising to see the emergence of vancomycin resistance. Fortunately, most of the vancomycin resistance genes detected are not associated with clinically important vancomycin-resistant enterococci (VRE). Nevertheless, we did find two patients with plasmid-encoded *vanA* genes associated with an *Enterococcus* species in their pre-FMT samples. Loss of detection of these plasmids in post-FMT samples supports the potential of FMT in achieving VRE decolonization, as previously reported in other studies.^51–55^

Repeated treatments of rCDI patients with vancomycin typically result in massive increases in the relative abundances of Enterobacteriaceae bacteria.^56,57^ This is not surprising as vancomycin is broadly active against the dominant phyla in the distal intestine, Bacteroidota and Bacillota, while sparing Pseudomonadota. Notably, we found that Enterobacteriaceae bacteria in the majority of rCDI patients, but not the donor, were associated with carriage of chromosomal and plasmid ESBL genes. FMT showed variable success in eliminating these genes. Interestingly, it was previously shown that post-FMT decrease in antibiotic resistance reservoir may take many months,^45^ and our study time frame may have been too short. It is also notable that the antibiotic treatment experienced by these patients was directed against *C. difficile* sparing Enterobacteriaceae. Multiple variables, including conditioning antibiotics that target Enterobacteriaceae carrying ESBL genes,^58,59^ different FMT dosing regimens,^58^ and even optimized donor selection strategies^37,38^ need to be studied in larger clinical trials. Proximity ligation shotgun metagenomics may be helpful in such studies to track the associations AMR gene-carrying plasmids and bacteriophages with clinically relevant pathobionts, such as members of the Enterobacteriaceae family.

## Materials and methods

### Ethics statement

The patients participating in this study received treatment with FMT under the US Food and Drug Administration ‘enforcement discretion’ policy for treatment of recurrent *C. difficile* infections not responding to standard therapies. The study of fecal specimens was approved by the University of Minnesota Institutional Review Board (STUDY00003519).

### Patients

Patients participating in this study were treated at the University of Minnesota *C. difficile* clinic using standardized capsule FMT, administered orally. Eligibility criteria included (1) documented recurrent *C. difficile* infections with multiple failures of antibiotic therapy alone over the preceding 12 months; (2) absence of underlying active inflammatory bowel disease; (3) no anticipated antibiotics within the next 3 months after FMT; (4) clinical informed consent for FMT; (5) research informed consent for fecal sample collection. The clinical demographics of the patient cohort is included in the Supplemental Materials (Table S1).

### Oral capsule FMT

The oral capsule FMT formulation MTP-101C, containing purified fecal microbiota from a single healthy donor, was manufactured using Good Manufacturing Practices protocols in accordance with the FDA-approved protocols (IND 15,071).^46^ Briefly, the volunteer donor, a 36-year-old male, eating an omnivorous but high fiber diet, was a participant stool donor in the University of Minnesota Microbiota Therapeutics Program.^26^ The stool was processed to extract the microbiota through a series of filtration and washing steps under nitrogen flow. The purified microbial fraction was then lyophilized using trehalose as the lyoprotectant.^46^ The freeze-dried microbiota was double encapsulated in hypromellose capsules (DRCaps, Lonza, USA). The patients stopped oral vancomycin 2 days before FMT. They were instructed to be on a clear liquid diet for at least 4 h before administration of FMT capsules and remain on clear liquids for an additional 2 h after FMT. The FMT consisted of four capsules containing a total of ~5 ×10^11^ live bacteria as determined by microscopy using the LIVE/DEAD™ *Bac*Light™ staining (ThermoFisher Scientific, Waltham, MA, USA).

### Fecal sample collection

Fecal samples were collected using Puritan HydraFlock swabs placed within 1 mL of the DNA/RNA preservative (Puritan Medical Products, Avantor, Radnor, PA). The samples were stored at −80°C until extraction.

### Library preparation and sequencing

Hi-C libraries were created using the Phase Genomics ProxiMeta Hi-C v4.0 Kit according to the manufacturer-provided protocol (Phase Genomics, Seattle, Washington).^60^ Briefly, intact cells were crosslinked using a formaldehyde solution, crosslinked DNA was digested with Sau3AI and MlucI restriction enzymes and proximity ligated with biotinylated nucleotides to create chimeric molecules. Streptavidin beads were used to pull down proximity ligated DNA molecules followed by an Illumina-compatible sequencing library prep. Separate aliquots of samples were processed for shotgun metagenomic sequencing as follows. DNA was extracted using a ZYMObiomics DNA miniprep kit (Zymo Research Corp, Irvine, California) and shotgun libraries were prepared using ProxiMeta library preparation reagents. Sequencing was done on an Illumina NovaSeq platform performed by Azenta Life Sciences (Burlington, MA, USA), generating PE150 read pairs for both Hi-C and shotgun libraries. Shotgun and Hi-C library stats are found in Supplementary Data (Table S2).

### Metagenome assembly, binning, and taxonomic annotation

Hi-C and shotgun metagenomic sequence libraries were processed with the Phase Genomics cloud-based bioinformatics platform. All shotgun libraries were filtered and trimmed for quality and normalized using fastp^61^ and then assembled with MEGAHIT^62^ using default options. Hi-C reads were then aligned to the assembly using BWA-MEM^63^ with the −5SP options specified, and all other options default. SAMBLASTER^64^ was used to flag PCR duplicates, which were later excluded from the analysis. Alignments were then filtered with SAMtools^65^ using the -F 2304 filtering flag to remove non-primary and secondary alignments. Metagenome deconvolution and binning of metagenome assembled genomes (MAGs) was performed using the ProxiMeta^66^ platform. MAG quality was assessed using CheckM^67^ and assigned taxonomic classifications with GTDB-Tk.^68^

Phage and plasmid contigs were identified from the metagenomic assemblies of each sample using VIBRANT^69^ and a proprietary plasmid identification pipeline, respectively. Briefly, to identify plasmid-derived contigs within metagenomic assemblies, contigs were aligned to a curated plasmid database using BLAST. Alignments were filtered to retain hits with ≥90% identity and ≥100 bp in length. For each contig, the best-matching plasmid was selected based on the product of identity and alignment length. Plasmid completeness was estimated as the fraction of the reference covered, and contamination was defined as overlapping alignments to the same reference region. Contigs were classified as plasmid-derived if they met one of the two criteria: (1) total aligned length ≥1,000 bp, or (2) ≥90% coverage of the reference in a single alignment.

Phage and plasmid bins were generated using the ProxiPhage^70^ viral binning method to produce more complete mobile element genomes. Antimicrobial resistance (AMR) genes were annotated using NCBI’s AMRFinderPlus^71^ (version 3.10.5) with the ‘—plus’ option specified, with all other options set to default. Mobile element-to-host associations were annotated using the ProxiPhage^70^ host association method. Host taxonomy assignments from GTDB-Tk^68^ were used for all phage-host, plasmid-host, and AMR-host connection-related analyses.

### Abundance, diversity, and statistical analyses

Abundance calculations for microbes, phages, and plasmids were calculated as follows. Forward shotgun reads were mapped to each corresponding assembly using bowtie2^72^ using the sensitive global assignment mode. Alignments were sorted, marked for PCR duplicates, and indexed using SAMTools.^65^ Reads per kilobase (RPK) for each bacterial, phage, or plasmid MAG was calculated as the total unique aligned read count divided by the corresponding genome length in kilobases. Transcripts per million (TPM) values were calculated for bacterial, phage, or plasmid fractions by dividing each observed RPK by the corresponding TPM scaling factor, the sum of RPK values divided by 1,000,000. Abundance ratios for engrafted bacteria and phages were calculated as the log base 2 of the ratio of TPM observed in a post-FMT sample divided by the TPM observed in the donor sample. AMR gene abundances were derived from the TPM observed for the assigned bacteria MAG.

Microbial and phage genomes were clustered into operational taxonomic units (mOTUs and vOTUs, respectively) to facilitate beta diversity and host-connectivity estimation across the complete cohort sample set. Microbial MAGs were filtered on CheckM quality metrics, completeness (≥80) and contamination (≤10). High-quality microbial MAGs were clustered using RabbitTClust^73,74^ using the ‘MiniHash’ sketch function, kmer size of 15, sketch size of 25,000, and a distance threshold of 0.05. Phage genomes were clustered following previously described methods for the MGV^75^ and IMGVR^76^ databases. All pairwise comparisons of phage genomes used BLAST^77^ with the following options: max_target_seqs 25,000, per_identity 90. Phages were clustered together if they met the following criteria: min_ani 95, min_qcov 0, min_tcov 85. Microbial and phage clusters were termed ‘engrafted’ if they were detected in the donor sample and one or more post-FMT samples, but not in any pre-FMT sample.

Microbial and phage alpha-diversity metrics were calculated as the inverse Shannon diversity index using relative abundances and the python scipy stats entropy library.^78^ Beta diversity comparisons were calculated as the Bray–Curtis similarity index using the python scipy spatial distance braycurtis library.^78^

Multiple group comparisons for alpha-diversity and beta diversity metrics were performed using one- or two-way analysis of variance (ANOVA) and Tukey’s honest significant differences post hoc analysis using the following python packages: scipy stats f_oneway,^78^ statsmodels anova_lm,^79^ and statsmodels multicomp pairwise_tukeyhsd.^79^

The microbe–phage interaction graph was constructed using the python NetworkX^80^ library and visualized with Cytoscape.^81^

## Supplementary Material

Revised Supplementary Table S2.xlsx

Supplementary Table S3.xlsx

Revised_Supplementary_Table_S1.docx

## Data Availability

The raw metagenomic data were deposited at Figshare: 10.6084/m9.figshare.28259270.
