# Supplementary material for: Use of proximity ligation shotgun metagenomics to investigate the dynamics of plasmids and bacteriophages in the gut microbiome following fecal microbiota transplantation
Source: Gut Microbes. 2025 Sep 15;17(1):2559019. doi: 10.1080/19490976.2025.2559019 (PMC12439552; doi:10.1080/19490976.2025.2559019)
Supplement: Revised_Supplementary_Table_S1.docx [file KGMI_A_2559019_SM8757.docx]

Supplementary Table 1. Clinical characteristics and demographics of the rCDI cohort.

|  | **rCDI patients (N = 30)** |
| --- | --- |
| Age (mean ± SD, years) | 60.4 ± 17.3 |
| Female sex | 24 (80.0%) |
| Charlson comorbidity index, median (range) | 3.0 (0, 8) |
| Underlying IBD | 2 (6.7%) |
| Number of prescription medications (mean ±  SD) | 4.6 ± 2.3 |
| History of abdominal surgeries | 14 (46.7%) |
| History of cholecystectomy | 6 (20.0%) |
| Acid blocking medications | 9 (30.0%) |
| Use of cholesterol medications | 9 (30.0%) |
| Use of metformin | 3 (10.0%) |
